# Prevalence of chronic cough in relation to upper and lower airway symptoms; the Skövde population-based study

**DOI:** 10.3389/fphys.2012.00251

**Published:** 2012-07-04

**Authors:** Mats Bende, Eva Millqvist

**Affiliations:** ^1^Department of Otorhinolaryngology, Central HospitalSkövde, Sweden; ^2^Department of Internal Medicine/Respiratory Medicine and Allergology, Sahlgrenska Academy, University of GothenburgGothenburg, Sweden

**Keywords:** chronic cough, epidemiology, odor intolerance, population-based, upper airways

## Abstract

The aim of this study was to determine the prevalence of chronic cough in relation to upper airway symptoms, in a cross-sectional, population-based epidemiological study. Another aim was to relate coughing to other explanatory variables and risk factors. A random sample of 1900 inhabitants from the age of 20, stratified for age and gender, was recruited. Subjects were invited for clinical examinations that included questions about general odor intolerance, respiratory symptoms, and smoking habits, and a smell identification test. In total, 1387 volunteers (73% of the sample) were investigated. The overall prevalence of self-reported chronic cough was 6.3% [95% confidence interval (CI): 5.0–7.6%]. Female gender, age, height, BMI, and smoking were significantly related to cough. Furthermore, nasal blockage, nasal secretion, sneezing, asthma, odor and cold air sensitivity, and aspirin intolerance also related to cough with statistical significance, indicating a close connection between chronic cough and upper airway symptoms. In keeping with other studies, this study demonstrates that chronic cough is a widespread problem in society, and is about twice as common in women than in men.

## Introduction

Cough, which is a common symptom among patients, often relates to upper and lower airway infections or heavy smoking (Chung and Widdicombe, 2004a,b). Both acute and chronic persistent cough can contribute to a significant reduction in quality of life measures in men and women (French et al., 2005). Cough is hard to treat and the most common reason for seeking medical help in the Western world (Chung and Pavord, 2008). Chronic cough that is not related to infection can be a diagnostic challenge (Chung and Pavord, 2008; Dicpinigaitis, 2011). After exclusion of pulmonary illnesses such as infection, cancer, foreign body aspiration, cystic fibrosis, alveolitis, asthma, and chronic obstructive pulmonary disease (COPD), the remaining explanations for chronic cough include medication with angiotensin-converting enzyme (ACE) inhibitor, gastroesophageal reflux disease (GERD), and post-nasal drip. Different clinics report different frequencies for both the causes of chronic cough and the success of treatment. In some cases, the cause of chronic cough cannot be explained. This unexplained cough is also known as chronic idiopathic cough (CIC), though there are widely varying opinions as to its existence. “Cough hypersensitivity syndrome,” a paradigm that accounts for unexplained cough, includes several groups of chronic cough patients, both those with symptoms that may indicate a reflux disease and patients with a general hypersensitivity toward for example environmental irritants (Morice, 2009; Chung, 2011; Millqvist, 2011).

This present study forms part of the Skövde population-based study (Johansson et al., 2003; Oberg et al., 2003; Bramerson et al., 2004; Akerlund et al., 2006), a larger cross-sectional investigation of a random adult sample regarding respiratory symptoms in Sweden. The main purpose of the study was to obtain epidemiological data regarding nasal polyps and smell dysfunctions. This report uses the original data to focus on the relationship between cough and the upper and lower airways. The aim is to determine the prevalence of chronic cough in relation to upper airway symptoms with a secondary aim to relate cough to other explanatory variables and risk factors.

## Materials and methods

### Participants

In the Skövde population-based study, a random sample of 1900 adults was drawn from the municipal directory. The sample was stratified by gender and age into seven groups, aged 20–29, 30–39, 40–49, 50–59, 60–69, 70–79, and 80+ years. An invitation to participate in the study was sent by mail, and the recipients were asked to phone the clinic for a study appointment. Non-responders received up to two reminders and, where possible, were also approached by telephone. A signed informed consent form was obtained from each participant. The study was carried out in accordance with the Helsinki Declaration and was approved by the Ethics Committee of the University of Gothenburg.

### Methods

All participants were examined using nasal rhinoscopy to identify nasal pathology, such as signs of inflammation, polyps, and septal perforations. Furthermore, smell disorders were registered by the Scandinavian Odor Identification Test (SOIT), as previously reported (Johansson et al., 2003; Oberg et al., 2003; Bramerson et al., 2004; Akerlund et al., 2006). Height and weight were measured and body mass index (BMI) was calculated. Medical history was gathered in a standardized manner by means of a structured interview. Cough was identified by the question: “Are you bothered by cough?” If the answer was “yes,” the respondent was asked to indicate whether symptoms were experienced daily, frequently, or occasionally, in order to differ between acute and more chronic symptoms. Subjects reporting daily or frequent cough were classified as having chronic cough. Information regarding relevant medical conditions, such as nasal blockage, nasal secretion, sneezing, asthma, and diabetes were obtained through other questions. Smoking habits were analyzed by several questions: “Have you *ever* smoked (almost every day during a year)?”, “Are you smoking *regularly* today?” and others about duration and amount of smoking to calculate the pack-years. Also, there were questions regarding self-reported sensitivity to irritating odors, cold air, and aspirin.

### Analyses

The significance of differences between variables was calculated with the unpaired *t*-test for continuously distributed variables, and the χ^2^ test for categorical variables. *P*-values < 0.05 were considered statistically significant. To identify factors related to daily and frequent symptoms of cough, a multiple logistic regression was fitted to the data. The following variables were selected in the model: age, gender, length, weight, BMI, SOIT, smoking, nasal blockage, nasal secretion, sneezing, asthma, diabetes, sensitivity to odors, aspirin (NSAID), and cold air, and meaningful interactions between these variables. To keep this model as simple and plausible as possible, stepwise selection (forward and backward) procedures were used. The significance level for entry and removal of a variable was set to 5%.

## Results

**Figure 1:**
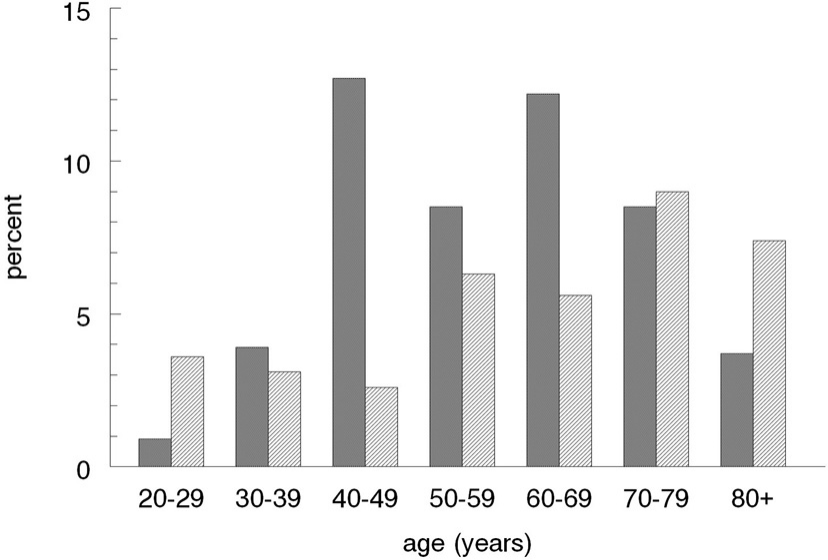
**Age and gender distribution in individuals with daily or frequent symptoms of cough.** Data presented in percentage of the total population. Dark bars = females.

Of the 1900 randomly selected individuals, 1387 (73%) presented for the clinical investigation and interview (Johansson et al., 2003). Fifty-four (3.9%) of these individuals reported daily symptoms of cough while 33 (2.4%) reported frequent and 102 (7.4%) reported occasional cough problems. The rest, 1198, (86.4%) reported no cough problems. In total, 87 individuals reported daily or frequent symptoms of cough, which gives a prevalence of 6.3% [95% CI (confidence interval): 5.0–7.6%]. Figure 1 illustrates the age and gender distribution of these subjects.

Table 1 presents a comparison between those with chronic cough symptoms (daily or frequent coughing) and the others reporting no cough symptoms (*n* = 1300) regarding age and gender, physiological data and symptoms. As shown, female gender, age, height, BMI and smoking were significantly related to cough. In addition, nasal blockage, nasal secretion, sneezing, asthma, odor and cold air sensitivity, and aspirin intolerance were also related to cough with statistical significance. The sense of smell measured with SOIT was not related to cough. In this test, those with cough identified one stimulus, the odorant with typical trigeminal stimulation, namely peppermint, less often than the other odorants, which did not stimulate the trigeminal nerve as much.

**Table 1:** **Anthropometric and clinical characteristics, as well as respiratory questionnaire results of individuals with daily or frequent symptoms of cough (*n* = 87) compared with others (*n* = 1300) in a randomly, age- and gender-stratified, selected population-based study of adults**.

**Variables**	**Individuals with cough**	**Others**	***p*-values**
Female gender (%)	63.2	51.1	<0.05
Age (years)	55.2	48.8	<0.001
Height (cm)	169	172	<0.01
Weight (kg)	77.2	75.7	n.s.
BMI (kg/m^2^)	27.0	25.5	<0.001
Nasal polyps (%)	4.6	2.6	n.s.
Ever smoked (%)	54.0	43.5	0.057
Regularly smoking (%)	31.0	15.9	<0.001
Smoking (pack-years)	10.6	6.0	<0.001
Daily nasal blockage (%)	20.7	8.8	<0.001
Daily nasal secretion (%)	18.4	5.4	<0.0001
Daily sneezing (%)	11.5	3.9	<0.01
Symptoms of asthma (%)	29.9	8.2	<0.0001
Diabetes (%)	8.2	4.2	0.052
Sense of smell (SOIT) correct answers of 16	13.5	13.8	n.s.
Not capable to identify peppermint (%)	28	12	<0.01
Odor sensitivity (%)	50.6	31.6	<0.001
Aspirin intolerance (%)	5.8	1.9	<0.05
Cold air sensitivity (%)	32.2	13.4	<0.0001

Multiple logistic regression analysis showed that asthma and sensitivity to odors were the most important predictors for cough. The odds ratio (OR) for cough in individuals with asthma was 3.67 (*p* < 0.0001), sensitivity to odors 1.67 (*p* < 0.05), nasal blockage 0.52 (*p* < 0.05), nasal secretion 0.34 (*p* < 0.01), regularly smoking 0.34 (*p* < 0.001), increasing age 0.15 (0.001), and increasing BMI 0.12 (*p* < 0.05). An analysis carried out separately for females and males separately, indicated that odor sensitivity was a more important factor in females than in males (OR = 4.0 against OR = 0), while in males asthma was more important (OR = 5.3 against OR = 3.3).

Among the 87 individuals, pack-year was not a strong predictor for cough. But, when analyzing pack-years among those who were, or had been, smokers (*n* = 611), those with cough had 19.7 pack-years (95% CI: 15.2–24.1) compared with subjects without cough who had 13.7 pack-years (95% CI: 12.5–15.0).

## Discussion

The current study, which examined the prevalence of cough in relation to upper airway symptoms and the relationship of coughing to explanatory variables and risk factors, demonstrated that chronic cough is common among adults and, further, that upper airway symptoms are overrepresented in chronic cough. Cough is regarded as chronic when it persists for more than 8 weeks though the definition of “chronic cough” varies in the literature (Morice et al., 2004) and in this study we chose to classify participants reporting daily or frequent problems with cough as having chronic cough. More specified questions had been valuable and may have influenced the results. Related factors of importance were other respiratory symptoms from both the upper and lower respiratory tract; cough was especially common in individuals with asthma and odor sensitivity. However, this study did not include information on how many of these adults with cough symptoms had sought medical care for their problems.

Though evident, the link between the upper and lower airways is often neglected. Following tradition, the ears, nose, and throat (ENT) and the pulmonary specialists have, respectively, taken care of the upper and lower airways, although the airways are one functional unit. A combination of neuroanatomical, neurophysiological, and functional findings shows the common origin and mechanisms in the airways, in vertebrates as well as in mammalians (Taylor et al., 1999). The upper and lower airways complement each other with regard to reflexes (the trigeminal reflexes initiated in the upper airways and the vagal reflexes evoked in the lower airways), which are regulated by the two main sensory nerves of the airways. The results of this current study emphasize the close connection between the upper and lower airways with regards to cough. The finding that coughers had significantly less ability to recognize the trigeminal stimulant peppermint which may indicate a disturbance of the airway sensory nervous system in accordance with the known augmented capsaicin cough sensitivity mediated by transient ion receptors on vagal and trigeminal nerves (Caterina et al., 1997; Dicpinigaitis and Alva, 2005).

Post-nasal drip syndrome (PNDS) has been suggested as one explanation of chronic cough (Irwin et al., 1990; Pratter et al., 1999) though this has been questioned (Morice, 2004; Sanu and Eccles, 2008). The explanation that nasal discharge is “dripping down from the nasopharynx,” and in that way induces chronic cough, is vague. PNDS may be caused by a mucus hypersecretory phenotype that develops following chronic exposure of the respiratory tract to particulate matter, allergens, irritants, and pathogens. The term PNDS has recently been replaced with a more general description, Upper Airway Cough Syndrome (Irwin et al., 2006), which indicates a more general relation between chronic cough and upper airway symptoms and is in keeping with the present findings. Perhaps, it is more simple and adequate to refer to PNDS as *chronic rhinosinusitis*?

Women are known to be more troubled by cough than men and to have increased cough sensitivity (Kastelik et al., 2002; Kelsall et al., 2009). In this study, especially in the ages between 40–50 years and 60–70 years, women reported more cough problems than men. This is in accordance with the findings of estrogen activation/sensitization of TRPV1 that may predispose the female sex to cough and increased cough sensitivity (Patberg, 2011).

Rhinitis and cough are two of the most common symptoms in clinical practice. Patients seeking medical care for either of these symptoms should always be asked whether they have any sign of symptoms in the other airway. Future research will likely teach us more about chronic cough; one way to enhance our knowledge in this field may be through an increased understanding of the upper airways' role in chronic coughing.

### Conflict of interest statement

The authors declare that the research was conducted in the absence of any commercial or financial relationships that could be construed as a potential conflict of interest.
